# Minimally invasive management of combined esophageal atresia with tracheoesophageal fistula and duodenal atresia: a comprehensive case report

**DOI:** 10.3389/fped.2023.1252660

**Published:** 2023-09-08

**Authors:** Eunyoung Jung

**Affiliations:** Division of Pediatric Surgery, Department of Surgery, Keimyung University School of Medicine, Dongsan Medical Center, Daegu, Republic of Korea

**Keywords:** esophageal atresia, tracheoesophageal fistula, duodenal atresia, laparoscopy, thoracoscopy, minimally invasive surgical procedures

## Abstract

A newborn presented with a rare combination of esophageal atresia with tracheoesophageal fistula (EA/TEF) and duodenal atresia (DA), which was successfully managed using minimally invasive surgical techniques. The patient was a 1-day-old male for whom passing a feeding tube was infeasible and who had a double bubble sign on radiography. The neonate underwent a thoracoscopic ligation of the tracheoesophageal fistula (TEF) and a laparoscopic duodeno-duodenostomy on the same day, resulting in stabilized vital signs. Ten days after the initial operation, a thoracoscopic esophago-esophagostomy was successfully performed. The patient demonstrated full feeding capability and normal weight gain after the surgeries. The co-occurrence of EA/TEF and DA is a rare and complex anomaly. This case indicates that minimally invasive techniques can effectively manage this condition.

## 1. Introduction

Esophageal atresia (EA) and duodenal atresia (DA) are rare congenital anomalies that occur independently. Their simultaneous manifestation is even rarer, accounting for approximately 2% of cases (1, 2). This dual anomaly presents unique challenges, instigating debates over surgical strategy, including the potential requirement for routine gastrostomy (3–6). The existing literature has mainly focused on cases managed with traditional open surgical techniques (6–9). However, this report aims to contribute a novel perspective, detailing a case of a newborn treated successfully for EA, tracheoesophageal fistula (TEF), and DA via staged, minimally invasive procedures with percutaneous gastrostomy. This manuscript was prepared following the CARE guidelines (https://www.care-statement.org).

## 2. Case description

**Figure 1:**
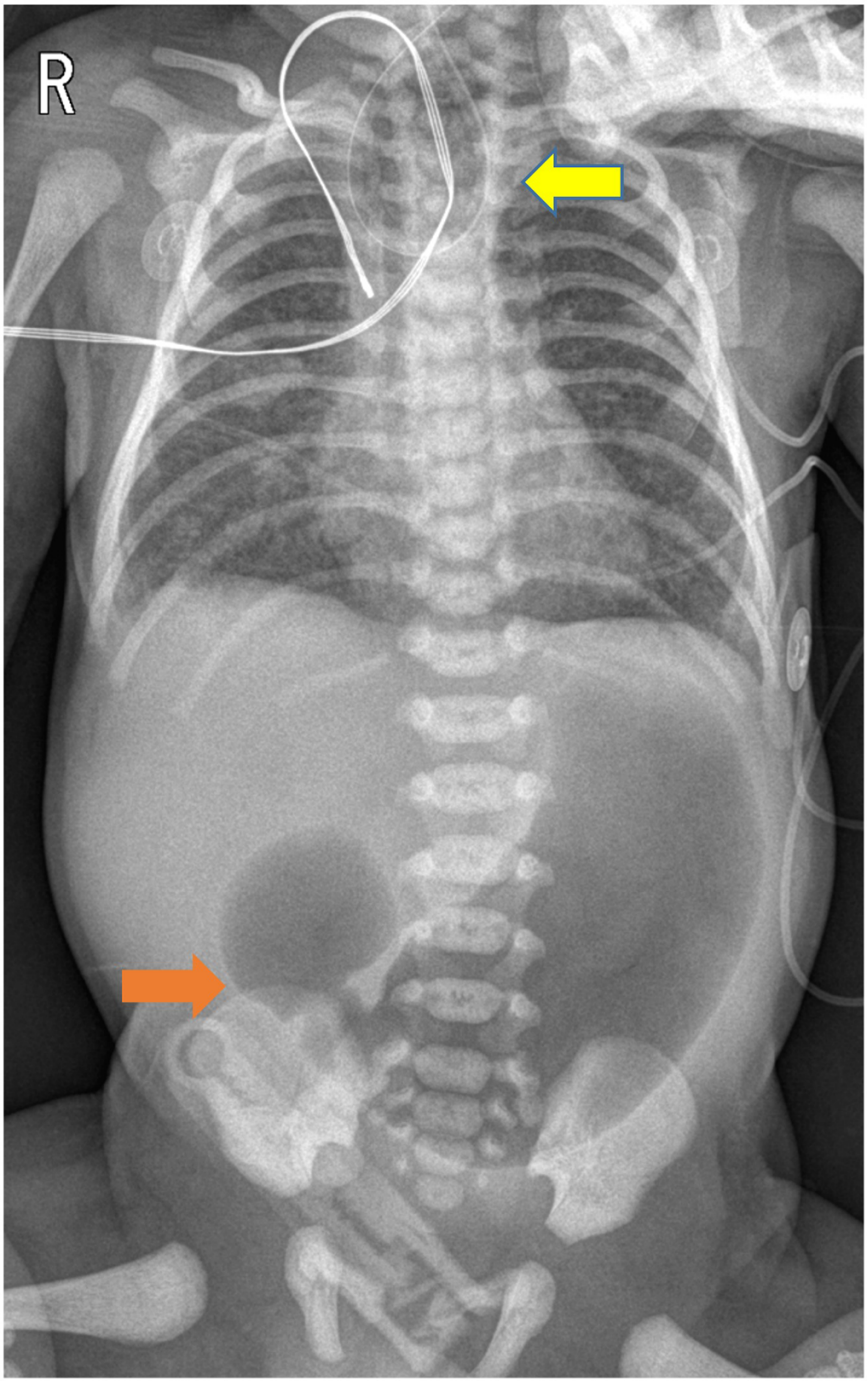
Chest and abdominal radiographs obtained post-orogastric tube insertion demonstrated a coiled orogastric tube (indicated by the yellow arrow) and the double bubble sign (indicated by the orange arrow).

**Figure 2:**
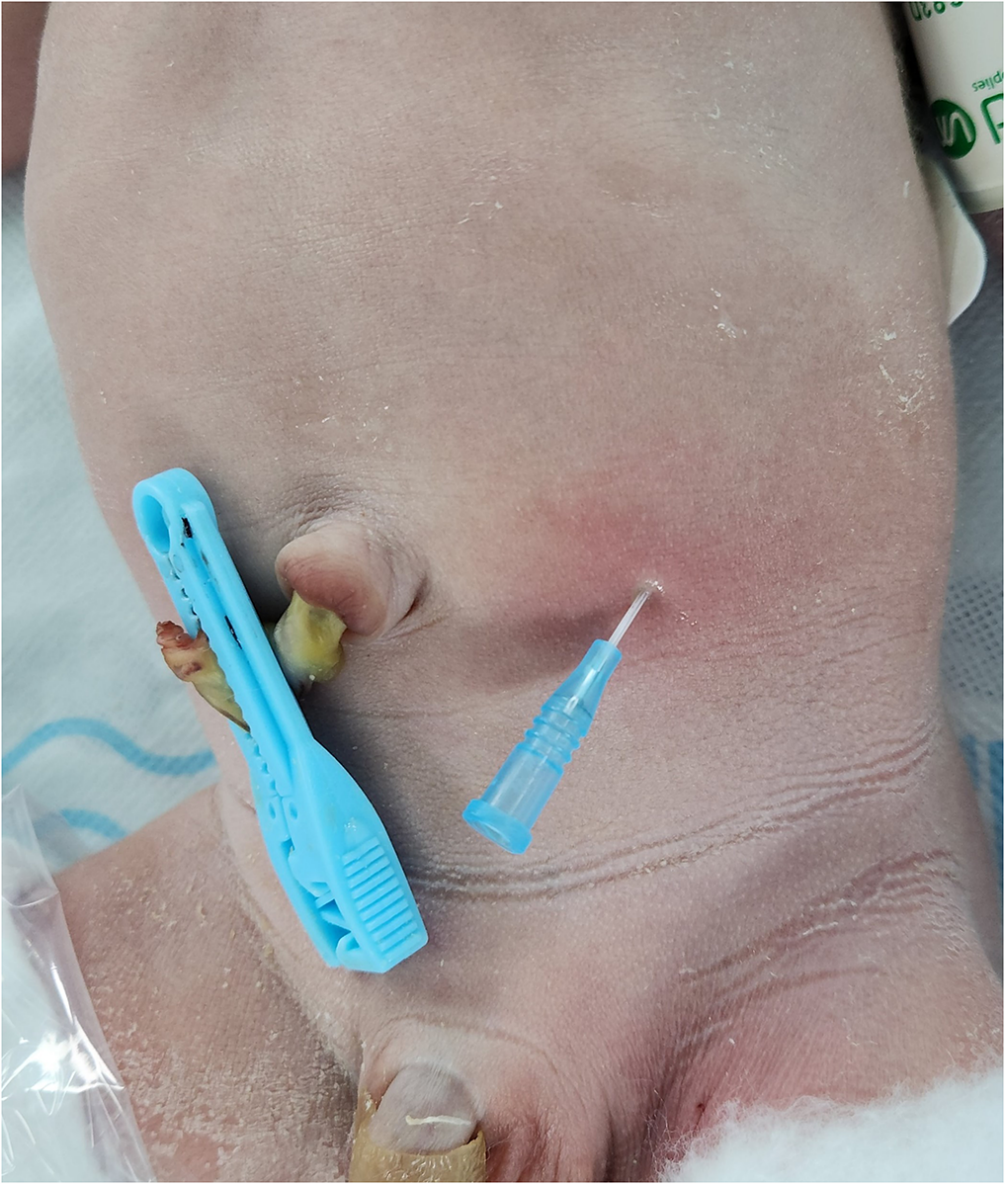
Before general anesthesia, a percutaneous 18G angiocatheter was placed in the stomach without imaging guidance, serving as a temporary gastrostomy.

**Figure 3:**
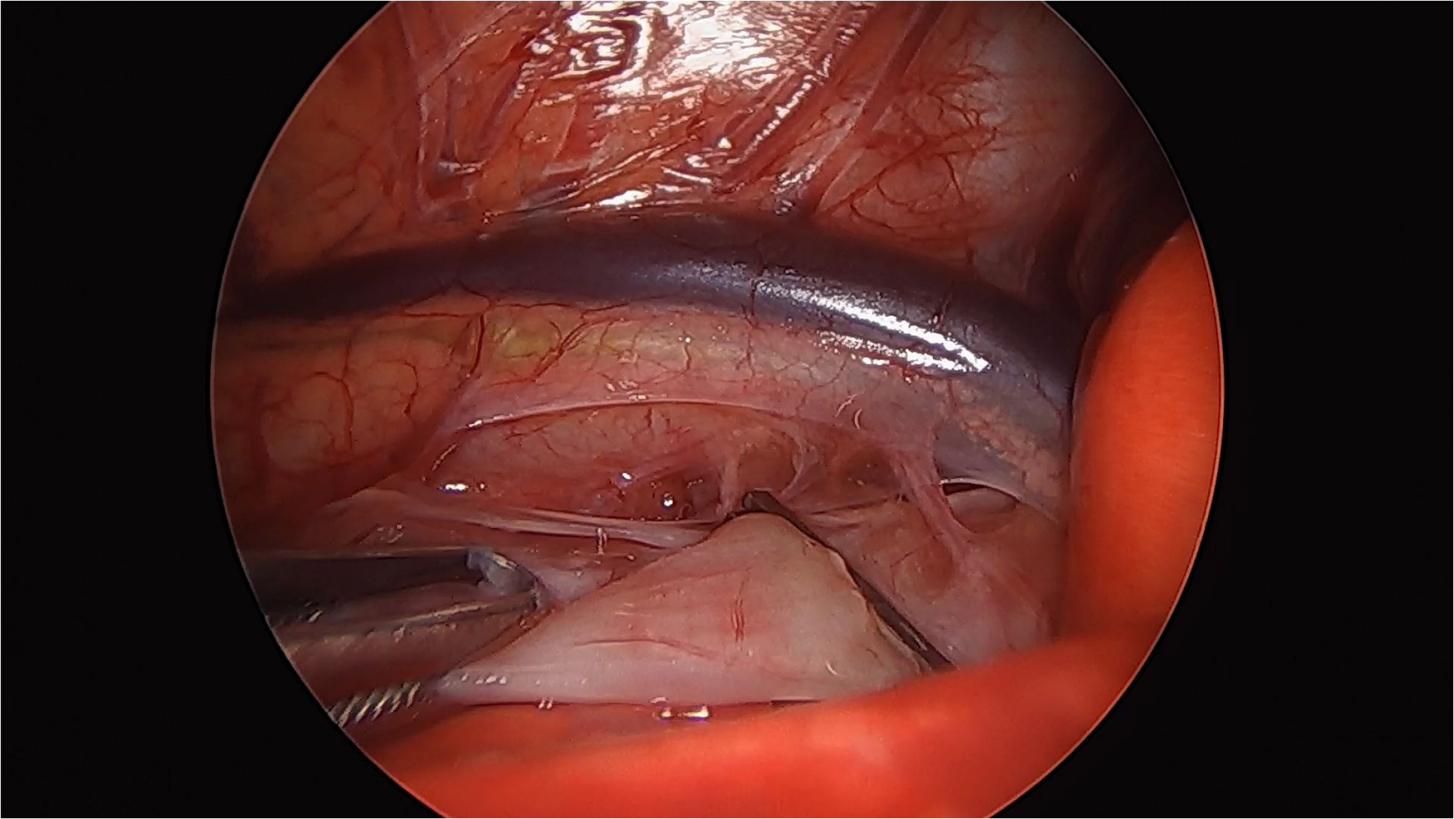
A singular 5-mm metal clip was used to close the tracheoesophageal fistula (TEF).

A 1-day-old male neonate was referred to the department after a failed attempt to pass a feeding tube shortly after birth. He was born via a planned cesarean section at a gestational age of 36 weeks and 3 days; the baby had a birth weight of 2,260 g. Polyhydramnios was diagnosed during the mother's pregnancy; however, the prenatal ultrasonography did not indicate the double bubble sign. The neonate's APGAR scores were 8 and 9 at 1 and 5 min, respectively. Immediate postnatal chest and abdominal radiographs revealed a coiled feeding tube in the esophageal pouch and a double bubble sign (Figure 1). These findings highly suggested the diagnoses of EA/TEF and DA. Although the newborn exhibited stable vital signs at birth, notable abdominal distension was observed without distal small- or large-bowel gas. With the onset of worsening abdominal distension and tachypnea, the team decided to operate on the day of birth. Before general anesthesia, gastric decompression was performed via percutaneous puncture with an 18G angiocatheter (Figure 2) in the operating room. With the patient supine, the distended stomach can be readily identified via visualization and percussion. This allows for the straightforward advancement of the catheter by percutaneous puncture without the need for imaging guidance. The catheter was kept in place until the completion of the thoracoscopic TEF ligation. Considering the preoperative echocardiography confirming the aortic arch's location on the left side, the surgical approach was from the right side, with the infant placed in a semi-prone position. The initial procedure involved thoracoscopic ligation of the TEF (Figure 3) using the conventional three-trocar technique. The operative time for thoracoscopic TEF ligation was 20 min. Following the TEF ligation, the infant's position was adjusted to supine for the succeeding procedure. A 5-mm umbilical port and two additional ports were inserted in the right and left abdomen. The angiocatheter for temporary gastrostomy was removed under the guidance of a laparoscope after decompression of the stomach and duodenum to facilitate good surgical view in laparoscopic DA repair. A laparoscopic duodeno-duodenostomy was conducted to manage type III DA. The time for the laparoscopic DA repair was 90 min. The patient demonstrated a preoperative venous CO_2_ level of 48.4 mmHg. The level was reduced to 39.0 mmHg immediately during the postoperative phase. During the intraoperative phase, the end-tidal carbon dioxide (ET_CO2_) level was consistently regulated at a mean of 32 mmHg (range: 30–36) during the thoracoscopic TEF ligation. Additionally, throughout the laparoscopic duodenostomy, the ET_CO2_ level was sustained at an average of 39 mmHg (range: 29–47). Postoperative vital signs, including respiration and arterial saturation, were stable. On postoperative day 10, with the patient's weight improved to 2,440 g, a thoracoscopic esophago-esophagostomy was performed via the previous trocar sites on the chest. The operative time was 140 min. Esophagography on postoperative day 5 showed no evidence of leakage or stricture. Consequently, feeding was initiated, and the patient was discharged on postoperative day 12, displaying full feeding capability. The patient's weight was 2.54 kg at discharge. At one month of life, he was 3.6 kg with full feeding. At 4 months, he was 7.3 kg that was normal growth with 60th percentile and without signs of esophageal stricture or reflux.

## 3. Discussion

The concurrent presentation of EA/TEF and DA is a challenging medical condition due to its infrequent occurrence. While the prenatal detection rates of EA and DA individually are over 70% (10, 11), their simultaneous detection is rare. Therefore, swift and precise diagnosis is crucial when both anomalies are identified postnatally, as seen in this case. Nevertheless, the rarity of these co-existing malformations means that comprehensive studies addressing optimal treatment strategies are limited, with most available information derived from case reports or series (3, 4). The primary surgical considerations include the necessity for gastrostomy (4) and the timing for addressing both anomalies – either through single- or multistage surgical procedures (3, 8).

Single-stage surgery might seem ideal; however, no definitive evidence suggests superior survival outcomes compared to multistage surgery. Conversely, hasty surgical interventions without carefully considering the neonate's overall condition could lead to decreased survival rates (3, 5). Table 1 summarizes previously reported cases of combined EA with TEF and DA. The surgical management options and survivals are variable, and all the reported cases were performed using open thoracotomy and laparotomy. Ideally, performing surgeries for EA with TEF and DA concurrently without the need for a gastrostomy seems optimal. Particularly, if one can utilize thoracoscopy and laparoscopy for the surgery, it would be even more advantageous. If the patient's condition is stable and the procedure is conducted by a well-experienced surgeon, combining the surgeries in one session seems best. However, to date, there are no reports on spontaneous repair via minimally invasive surgery. Moreover, spontaneous repairs performed thoracotomy and laparotomy were reported to have a higher mortality compared to multistage repairs (3). Therefore, a tailored management plan that fits the patient's situation is essential. For multistage procedures, most literature indicates that surgery for esophago-esophagostomy, with or without gastrostomy, is usually conducted first (5–9). Although no literature explicitly states the reason, one can infer that for open thoracotomy, since one needs to make an incision for esophageal atresia with tracheoesophageal fistula ligation anyway, performing esophago-esophagostomy directly after closing the thoracotomy, instead of conducting another laparotomy for duodeno-duodenostomy, seems more practical. However, in some cases, there are concerns about complete duodenal obstruction due to duodenal atresia, leading to the implementation of a gastrostomy (8). In our case, since the duodenal atresia surgery was performed as a first stage operation, there was an effect of gastric decompression through duodeno-duodenostomy, eliminating the need to maintain a gastrostomy post-surgery.

**Table 1:** Summary of all studies for the type and surgeries for combined esophageal atresia and duodenal atresia.

	Period	Cases (*N*)	Mean GA^1^ (range)	Mean BW^2^ (range)	Type C EA (*N*)	Gastrostomy	Simultaneous repair	Staged repair	EE first^3^	Mortality
Lee et al. (7)^a^	1989–2006	7	35 (32–40)	1,871 (1,100–2,600)	7/7 (100%)	3/7 (43%)	5/7 (71%)	1 (14%)	1/1	29%
Nabzdyk et al. (8)	2010–2012	3	34 (33–35)	1,822 (1,388–2,153)	3/3 (100%)	3/3 (100%)	0/3 (0%)	3/3 (100%)	3/3	0%
Fragoso et al. (12)^b^	1965–2012	10	35 (32–37)	2,240 (1,660–3,120)	10/10 (100%)	8/10 (80%)	8/10 (80%)	2/10 (20%)	1/2	50%
Miscia et al. (5)^c^	2000–2019	5	35 (33–36)	1,911 (1,350–2,365)	4/5 (80%)	1/5 (20%)	4/5 (80%)	1/5 (20%)	1/1	0%
Cao et al. (9)	2015–2018	4	39 (38–40)	2,748 (2,310–3,400)	4/4 (100%)	1/4 (25%)	3/4 (75%)	1/4 (25%)	1/1	0%
Ein et al. (3)^d^	1971–2000	24	35 (30–39)	2,100 (1,130–3,450)	17/24 (71%)	19/24 (80%)	7/24 (29%)	16/24 (67%)	unknown	25%
Dave et al. (6)^a^	1974–2003	10	36 (34–40)	2,317 (1,835–2,830)	9/10 (90%)	6/10 (60%)	4/10 (40%)	5/10 (50%)	5/5	10%
Spitz et al. (4)^e^	1964–1978	18	36 (30–42)	2,000 (1,200–2,800)	16/18 (89%)	5/18 (28%)	4/18 (22%)	8/18 (44%)	3/8	67%

GA, gestational age, weeks; BW, birth weight, gram; EE first, esophago-esophagostomy as a first operation in staged repaired patients.

^a^ One case expired without treatment.

^b^ No information for mortality cases, only analyzed 10 survival case among 20 combined EA and DA.

^c^ One case of staged repair is due to delayed diagnosis of duodenal web.

^d^ Mortality is 12% In type C patients, and 57% in pure EA patients.

^e^ Four cases were not treated.

In this case, persistent abdominal distension and tachypnea soon manifested despite the patient's initial stability. In instances of TEF without DA, stomach decompression might not be essential as stomach gas can transit through the distal gastrointestinal tract. However, with complete obstruction due to DA, continuous gastric distension could lead to irreversible lung damage from gastric aspiration via the TEF, necessitating emergency TEF ligation.

Although some studies advocate DA correction first before TEF ligation, if air entry into the stomach through TEF can be controlled with appropriate management of tidal volume, such delicate control is often challenging. Furthermore, some reports highlight the risk of gastric perforation during mechanical ventilation (13, 14). Therefore, beginning with TEF ligation can help stabilize the patient's airway. Similarly, Spitz et al. (4) recommended the creation of a wide gastrostomy following EA/TEF repair.

The sequence of operations addressing EA/TEF and DA remains undetermined. While other reports performed fluoroscopy-guided percutaneous gastrostomy (15), an 18G angiocatheter for stomach decompression was utilized as an alternative to initial gastrostomy without imaging guidance in the current case, facilitating uncomplicated thoracoscopic TEF ligation. This shortens the procedure time for TEF ligation and minimizes the gastrostomy scar. This strategy prevented severe gastric dilatation and aspiration from excessive tidal volume during intraoperative ventilation, providing a clear surgical field view for the subsequent DA operation.

In situations where the patient's condition could rapidly deteriorate if a time-consuming laparoscopic duodeno-duodenostomy is performed first, it is deemed safe to perform TEF ligation as soon as possible.

With advancements in neonatal surgery, anesthesia, and care, primary simultaneous repair has become an option. However, definitive evidence suggesting its superiority over staged operations is lacking. Ein et al. (3) reported a 75% survival rate in 24 cases of combined EA/DA over 30 years, with multistage operations unexpectedly showing better survival rates than single-stage procedures.

While single-stage surgeries may seem advantageous, the extended durations of anesthesia and surgery pose significant safety risks for neonates. Therefore, insisting on single-stage surgery without considering the potential negative impact on the newborn's outcome is unwarranted.

In conclusion, the co-occurrence of EA/TEF and DA is rare, necessitating a treatment approach that adapts to the individual patient and the resources available at the treating center. This report details a recent successful experience with a two-stage operation conducted without gastrostomy and utilizing minimally invasive surgical techniques. This approach underscores the evolving potential for neonatal treatment strategies in managing such complex cases.

## Data Availability

The original contributions presented in the study are included in the article, further inquiries can be directed to the corresponding author.
